# The Glyoxysomal Protease LON2 Is Involved in Fruiting-Body Development, Ascosporogenesis and Stress Resistance in *Sordaria macrospora*

**DOI:** 10.3390/jof7020082

**Published:** 2021-01-26

**Authors:** Antonia Werner, Kolja Otte, Gertrud Stahlhut, Leon M. Hanke, Stefanie Pöggeler

**Affiliations:** Institute of Microbiology and Genetics, Department of Genetics of Eukaryotic Microorganisms, Georg-August-University Göttingen, Grisebachstr. 8, 37077 Göttingen, Germany; ajakobs1@gwdg.de (A.W.); koljalukas.otte@stud.uni-goettingen.de (K.O.); gstahlh@gwdg.de (G.S.); leonmarius.hanke@stud.uni-goettingen.de (L.M.H.)

**Keywords:** Lon protease, microbodies, peroxisomes, glyoxysomes, fruiting-body development, stress resistance, *Sordaria macrospora*

## Abstract

Microbodies, including peroxisomes, glyoxysomes and Woronin bodies, are ubiquitous dynamic organelles that play important roles in fungal development. The ATP-dependent chaperone and protease family Lon that maintain protein quality control within the organelle significantly regulate the functionality of microbodies. The filamentous ascomycete *Sordaria macrospora* is a model organism for studying fruiting-body development. The genome of *S. macrospora* encodes one Lon protease with the C-terminal peroxisomal targeting signal (PTS1) serine-arginine-leucine (SRL) for import into microbodies. Here, we investigated the function of the protease SmLON2 in sexual development and during growth under stress conditions. Localization studies revealed a predominant localization of SmLON2 in glyoxysomes. This localization depends on PTS1, since a variant without the C-terminal SRL motif was localized in the cytoplasm. A ΔSmlon2 mutant displayed a massive production of aerial hyphae, and produced a reduced number of fruiting bodies and ascospores. In addition, the growth of the ΔSmlon2 mutant was completely blocked under mild oxidative stress conditions. Most of the defects could be complemented with both variants of SmLON2, with and without PTS1, suggesting a dual function of SmLON2, not only in microbody, but also in cytosolic protein quality control.

## 1. Introduction

In fungi, microbodies comprise single membrane bound organelles, including peroxisomes, glyoxysomes and Woronin bodies, which play important roles during developmental processes [1,2]. Woronin bodies are fungal specific organelles carrying mainly the HEX1 protein and are required for plugging of septal pores after wounding of hyphae [3,4]. Peroxisomes and glyoxysomes contain enzymes required for the catabolism of fatty acids (FA), biotin and secondary metabolite biosynthesis [2,5,6]. Folded proteins are imported into the lumen of microbodies when they possess a so-called peroxisomal targeting signal (PTS). These are short amino acid motifs found either at the C-terminus (PTS1), near the N-terminus (PTS2) or at internal regions (PTS3). PTS1 and PTS3 sequences are recognized by the cytoplasmic receptor Pex5p, while peroxisomal matrix proteins harboring PTS2 are recognized by the receptor Pex7p [5,6].

Usually, filamentous ascomycetes carry at least two distinct types of microbodies, Woronin bodies and peroxisomes or glyoxysomes. As initially defined by DeDuve [7], peroxisomes contain oxidases producing H_2_O_2_ as well as catalases decomposing the toxic hydrogen peroxide. Glyoxysomes harbor the key enzymes of the glyoxylate cycle, but typically no catalases [8]. Unlike most filamentous ascomycetes, members of the order Sordariales such as *Neurospora crassa* and *Podospora anserina* possess no real peroxisomes with catalase activity [8]. In *N. crassa* and *P. anserina*, the first step in fatty acid β-oxidation is catalyzed by an acyl-CoA dehydrogenase instead of an H_2_O_2_-producing acyl-CoA oxidase. Furthermore, the hydrogen peroxide generating urate oxidase is localized in the cytosol and not in microbodies. Therefore, true peroxisomes are missing in this clade of fungi; instead of peroxisomes, they possess glyoxysomes [8,9].

*Sordaria macrospora* is a coprophilous filamentous ascomycete and a close relative of *N. crassa* and *P. anserina* within the order Sordariales [10]. The fungus is used for more than 25 years as a model to study fruiting-body development and meiosis [11,12,13]. Recently, we demonstrated that the selective degradation of superfluous or damaged glyoxysomes by selective and non-selective autophagy is required for proper growth and sexual development in *S. macrospora* [14,15].

This study raises the question of whether and how a disturbed glyoxysomal protein quality control affects developmental processes in *S. macrospora*. Frequently, proteins entering the lumen of microbodies become useless due to detrimental modifications or improper folding. In these cases, ATP-dependent Lon-proteases in the lumen of the microbodies remove abnormal proteins by degradation or by mediating refolding with their chaperone activity [16]. The name of the protease Lon derived from a long form UV-irradiation sensitive *Escherichia coli* mutant resulting in the isolation of the first AAA+ (ATPase associated with diverse cellular activities) protease La [17,18]. Lon proteases form ring-shaped homo-oligomers. Each subunit possesses a substrate binding domain in the N-terminal region, a central ATPase domain with Walker A and B ATP-binding motifs and a carboxyl-terminal protease domain [19]. In eukaryotes, Lon proteases have been described as the main protease for degradation of damaged proteins in microbodies, mitochondria and chloroplasts [20]. Defects in the plant peroxisomal LON2 protease lead to the failure of degradation of obsolete glyoxylate cycle proteins, a reduced matrix protein import, enlarged peroxisomes, auxin resistance, and an induced pexophagy [21]. The mammalian peroxisomal LONp protease has been shown to control the matrix import of PTS1 proteins [22]. In the methylotrophic yeast *Hansenula polymorpha*, deletion of the peroxisomal protease Lon gene *pln* caused a slight increase in the number of peroxisomes, an increase of protein aggregates in the organelles, and resulted in enhanced oxidative stress [23]. Similarly, the deletion of the peroxisomal *lon* gene resulted in filamentous ascomycetes in enhanced oxidative stress, catalase- peroxidase accumulation in peroxisomes, a decrease in conidia production and vegetative growth [24,25].

In the present study, we aimed to provide insight into the role of glyoxysomal Lon-dependent protein quality control in the fruiting-body development of fungi. We identified the *Smlon2* gene coding for a glyoxysomal Lon protease with a C-terminal PTS1 targeting sequence in *S. macrospora*. We show that SmLON2 mainly localizes to microbodies and that this localization depends on the C-terminal PTS1. The deletion of *Smlon2* resulted in impaired fruiting-body development and ascospore production, as well as an expansion of aerial mycelium. Moreover, the deletion mutant was hypersensitive to exogenously applied H_2_O_2_. All mutant defects were complemented with the wild type *Smlon2* gene, while some were also complemented by a mutated gene version coding for a protein lacking the C-terminal PTS1. These data suggest that SmLON2 is not merely involved in microbody protein homoeostasis, but also has functions in the cytoplasm.

## 2. Materials and Methods

### 2.1. Strains, Media and Growth Conditions

All strains used in this study are presented in Table 1. *Escherichia coli* strain MACH1 (Thermo Fisher Scientific, C862003, Waltham, MA, USA) was used for the cloning and propagation of recombinant plasmids (Table 1) using standard culture conditions [26]. Homologous recombination was performed to generate recombinant plasmids in *Saccharomyces cerevisiae* strain PJ69-4A [27,28] and clones were selected for uracil prototrophy. *Sordaria macrospora* strains were transformed with recombinant plasmids (Table S1) as described previously [29,30]. Selection of transformants was performed on media containing nourseothricin-dihydrogen sulphate (50 µg mL^−1^, nat) (Jena Bioscience GmbH, AB-102XL, Jena, Germany) or hygromycin B (110 U mL^−1^, hyg) (Merck, 4400051-10MU, Kenilworth, NJ, USA). *S. macrospora* strains were grown at 27 °C on liquid or solid biomalt maize medium (BMM), or Sordaria Westergaard (SWG) fructification medium under continuous light conditions [31,32,33]. For phenotypic analysis under different stress conditions, the temperature was changed to 30 °C or SWG medium was supplemented with 2.5 mM 3-amino-1,2,4-triazole (3-AT; Sigma-Aldrich, A8056, Taufkirchen, Germany) or 0.005–0.02% H_2_O_2_. To change the carbon source, glucose of the SWG medium was reduced to 0.5% and supplemented with 0.15% oleic acid (OA, Sigma-Aldrich, O1008, Taufkirchen, Germany) and 0.05% Nonidet^®^ P40 (NP-40, AppliChem, A2239, Darmstadt, Germany). For the analysis of the growth rate, 30-cm race tubes were filled with 25 mL of solid SWG medium or stress-inducing medium and inoculated with a mycelial plug of 0.5 cm in diameter at one end. The growth front was marked every 24 h for 5 consecutive days of 3 replications in triplets. For phenotypic analysis and light microscopy, *S. macrospora* strains were grown on cellophane or glass slides covered with solid SWG or BMM medium at 27 °C with continuous light. The growth period differed between 3–10 days depending on the developmental stage that was analyzed. Primary transformants were crossed with the color spore mutant fus1-1 [34] to obtain single spore isolates (ssi), as described previously [35].

### 2.2. Generation of Plasmids

All template plasmids and primers used for PCR amplifications are listed in Tables S1 and S2, respectively. Primers were synthesized by Sigma-Aldrich Chemie GmbH (Taufkirchen, Germany). Plasmids generated for this study were constructed by homologous recombination in *S. cerevisiae* [27]. Plasmid pTagRFP-T_nat expressing free TagRFP-T (tRFP) was generated by amplifying the *ccg1* promoter of *Neurospora crassa* from plasmid pHAN1 [37] with primer pair pRSccg1/Pccg1_RFP, the *tagrfp-t* ORF from plasmid pAL5Lifeact [38] with primer pair RFP-f/RFP-r-trpC and the *trpC* terminator of *Aspergillus nidulans* from plasmid p1783-1 [39] with primer pair TrpC_F/pRS426GFPrev. The three fragments were cloned into vector pRS_nat [40] by homologous recombination in *S. cerevisiae*.

To localize the full-length SmLON2, an N-terminal eGFP-tagged and TagRFP-T-tagged variant of *Smlon2* under control of the native promoter and terminator was constructed. The *Smlon2* promoter with 29-bp overhang to the pRS-vector and the fluorescence gene was amplified from wild type (WT) gDNA using primer pair lon2-ko-5f/lon2-p-3r or lon2-ko-5f/Smlon2P_trfp. The *egfp* and *tagrfp-t* were amplified from plasmids p1783-1 and pTagRFP-T_nat with primer pairs GFP-f/egfp-r-Smlon2 and RFP-f/trfp-r_Smlon2, respectively. To allow a continuous open reading frame, both fragments harbor no stop codon and contain a 29-bp overhang to the *Smlon2*. The coding sequence of *Smlon2* ORF together with the native terminator was amplified using WT gDNA as template and primer pair Smlon2-f-ATG/lon2-ko-3r. This fragment comprises no start codon and a 29-bp overhang to the neighbored sequence. All fragments were combined into the *Xho*I-linearized vector pRS-nat and for the *egfp* fusion construct also in pRS-hyg [41] and resulted in N-terminal eGFP- or tRFP-tagged versions of SmLON2 in plasmid pegfp-Smlon2 (nat/hyg) and ptrfp-Smlon2 (nat), respectively. The version of *Smlon2* without the three amino acids serine-arginine-leucine (SRL) (pegfp-Smlon2ΔSRL) was amplified as described above by using primer combination lon2-ko-5f/lon2-SRLr2 and pegfp-Smlon2 as template DNA. The resulting fragment is characterized by the *Smlon2* promoter, *egfp* and *Smlon2* ORF without the SRL sequence. The terminator fragment was amplified with primer pair lon2-SRLf/lon2-ko-3r using the same template. DNA sequencing of the plasmids was performed by Seqlab Sequence Service Laboratories GmbH (Göttingen, Germany).

### 2.3. Construction of the Knockout Strain ΔSmlon2

To delete the *Smlon2* gene in *S. macrospora*, the knockout construct was generated by homologous recombination in yeast PJ69-4A. The 5′-(1062 bp fragment) and 3′-(983 bp fragment) flanking regions of *Smlon2* were amplified from WT gDNA using the primer pairs lon2-ko-5f/lon2-ko-5r-hph and lon2-ko-3f-hph/lon2-ko-3r carrying 29 bp overhangs for the pRS426 vector and the hygromycin B resistance (*hph*) cassette, respectively. The *hph*-cassette was amplified from plasmid pRS-hyg with the primers hph-f/hph-r. Subsequently, the three amplicons were co-transformed together with the *Eco*RI/*Xho*I-linearized vector pRS426 into the yeast strain PJ69-4A. The resulting plasmid pSmlon2-KO was used as template to amplify the 3469 bp deletion cassette with the primer pair lon2-ko-5f/lon2-ko-3r, containing the 5′ and 3′ flanking region of *Smlon2* and the *hph* cassette. The amplicon was desalted, and *S. macrospora* WT strain was transformed with the deletion cassette to replace the *Smlon2* ORF by the *hph* cassette by homologous recombination [29]. Primary transformants were crossed with the color spore mutant fus1-1 (S23442) [34] and single-spore isolates (ΔSmlon2) with hygromycin resistance were selected [35]. The absence of the *Smlon2* gene and the integration of the *hph*-cassette at the desired locus was verified using primer pairs lon2-ko-v5f/tC1_0 (1400 bp), h3_0/lon2-ko-v3r (1200 bp) and lon2-s3-f/lon2-s4-r (1100 bp). Deletion of *Smlon2* was further verified by Southern hybridization. Therefore, 30 µg of WT and ΔSmlon2 gDNA were hydrolyzed with *Xba*I. The digested gDNA was separated on an 1% agarose gel. A capillary blot with nylon membrane (GE Healthcare, Amersham RPN303B, Boston, MA, USA) was performed overnight at RT. The 630-bp *Smlon2* probe was amplified with primer pair lon2-ko-v3f/lon2-ko-v3r from *S. macrospora* gDNA. The probe was labeled with the Amersham AlkPhos Direct Labelling and Detection Kit (GE Healthcare, Amersham RPN3680, Boston, MA, USA). Detection was performed according to the manufacturer’s manual.

### 2.4. Quantitative Analyses of S. macrospora Strains

All quantitative analyses of *S. macrospora* strains were done using SWG medium. Counting of perithecia per cm^2^ was performed after 8 days of growth with the Digital Microscope VHX-500F (Keyence, Germany) with 10 independent measurements of three independent experiments (*n* = 30). Discharged ascospores were analyzed after 10 days of growth by washing off the ascospores from the lid of the dish and counting the ascospores using a Thoma cell counter chamber (W. Schreck, Hofheim, Germany). For each strain, the experiment was performed five times for three biological replicates (*n* = 15). All data presented are means with standard deviation. To test whether two data sets differed significantly; a two-tailed Student’s t-test for pair-wise statistical analysis was performed.

### 2.5. Light and Fluorescence Microscopy

For the investigation of sexual structures and hyphae, *S. macrospora* strains were grown for two to ten days on SWG covered glass slides and documented with a VHX-500F Digital Microscope (Keyence, Germany) or AxioImager M1 microscope (Zeiss, Jena, Germany) with differential interference-contrast (DIC). Images were captured using a Photometrix CoolSNAP HQ camera (Roper Scientific, Photometrics, Tucson, AZ, USA). Image processing was done using ZEISS ZEN Digital Imaging (version 2.3; Zeiss).

For fluorescence microscopic analysis, *S. macrospora* strains were grown on solid medium on top of a piece of cellophane (2 cm × 2 cm) in petri dishes at 27 °C for the indicated hours or days. For the detection of the eGFP signal chroma filter set 49002 and for DsRED/TagRFP-T chroma filter set 49005 (exciter ET470/40x, ET545/30x, emitter ET525/50 m, ET620/60 m and beamsplitter T495lpxr, T570lp) were used.

### 2.6. Protein Domain Analysis and Phylogeny

Domains were predicted with programs SMART (http://smart.embl-heidelberg.de/) [42] and Prosite (https://prosite.expasy.org/) [43]. The mitochondrial target sequence was predicted with the program MitoFates (http://mitf.cbrc.jp/MitoFates/cgi-bin/top.cgi) [44]. Schematic illustration was designed in the same relation regarding the amino acids, which is indicated in the figure. Multiple sequence alignments of protein sequences and a neighbor joining phylogenetic analysis was performed with MAFFT version 7 [45]. A bootstrap analysis was conducted with 1000 iterations to test the tree for statistical significance. The tree was displayed with Archaeopteryx.js [46].

## 3. Results

### 3.1. S. macrospora Encodes Two Lon Proteases

**Figure 1:**
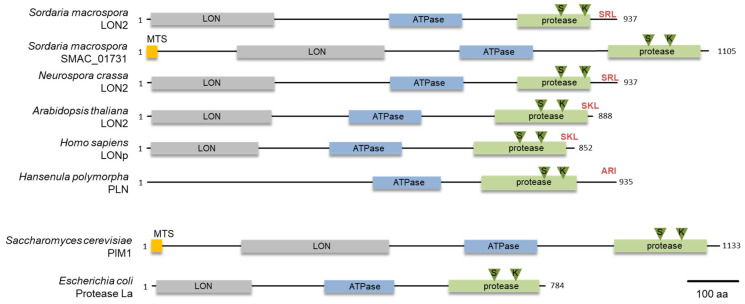
Domain organization of Lon proteases from fungi, plants, animals and bacteria. Domains were predicted with programs SMART [42] and Prosite [43], the mitochondrial targeting signal (MTS) indicated in orange was predicted with the program MitoFates [44]. The N-terminal Lon domain is indicated in grey, the ATPase domain is indicated in blue and the protease domain is depicted in green. The PTS1 sequence is shown in red and the S and K residues of the protease catalytic dyad is marked by green triangles. An N-terminal LON domain was not predicted for PLN of *H. polymorpha*. Accession numbers of the proteins are the following: *S. macrospora* SmLON2 (KAA8632750.1), *S. macrospora* SMAC_01731 (XP_024511168.1) *N. crassa* LON2 (NCU08303, XP_962516.1), *A. thaliana* LON2 (NP_568675.1), *Homo sapiens* LONp (NP_113678.2), *H. polymorpha* PLN (ABB88892.1), *Saccharomyces cerevisiae* PIM1 (CAA84841) and *E. coli* La (P0A9M0).

To identify a glyoxysomal LON protease in *S. macrospora*, we conducted a database search with the peroxisomal LON protease of *H. polymorpha* (ABB88892.1) as a bait protein. We used this protein as bait protein because it is a well characterized fungal peroxisomal protease [23]. This search revealed the presence of two putative *lon* genes, *SMAC_00912* and *SMAC_01731*, in the genome of *S. macrospora* [47]. The deduced amino acid sequence had 43% and 33% identity, respectively, with the *H. polymorpha* PLN protein. Both proteins contain the three conserved domains of Lon proteases, namely an N-terminal substrate binding LON-domain, a central AAA+ domain and the C-terminal proteolytic domain (Figure 1) [48]. The protein sequence of *SMAC_00912* (with PTS1) ends with C-terminal tripeptide SRL, which fits the PTS1 consensus sequence (S/A/C) (K/R/H) (L/M) of peroxisomal proteins [49], while the program MitoFates predicted an N-terminal mitochondrial targeting signal (MTS) (probability 0.981, http://mitf.cbrc.jp/MitoFates/cgi-bin/top.cgi) for SMAC_01731 (with MTS) [44]. From these data, we hypothesized that *SMAC_00912* encodes a glyoxysomal enzyme. The gene designated *Smlon2*. SmLON2 is a protein of 937 amino acids with calculated pI of 6.11 and molecular mass of 101.5 kDa. Like peroxisomal Lon proteases from plants, animals and fungi, SmLON2 can be classified as A-type Lon with a predicted large N-terminal domain (Lon-domain aa 11–256), a central AAA+ domain including typical Walker A and B motifs (aa 483–629) and a proteolytic domain (aa 734–921) with a Ser-Lys catalytic dyad (S827 and K870). BLASTP analyses with SmLON2 protein sequence revealed the high percentage of sequence identity with a putative glyoxysomal LON proteases of *N. crassa* (94.13%, NCU08303) (Figure 1). A phylogenetic analysis of SmLON2 and orthologs from fungi, plants, animals and bacteria revealed that SmLON2 clusters with peroxisomal Lon proteases from other organisms (Figure S1).

### 3.2. SmLON2 Localizes Predominantly to Microbodies

**Figure 2:**
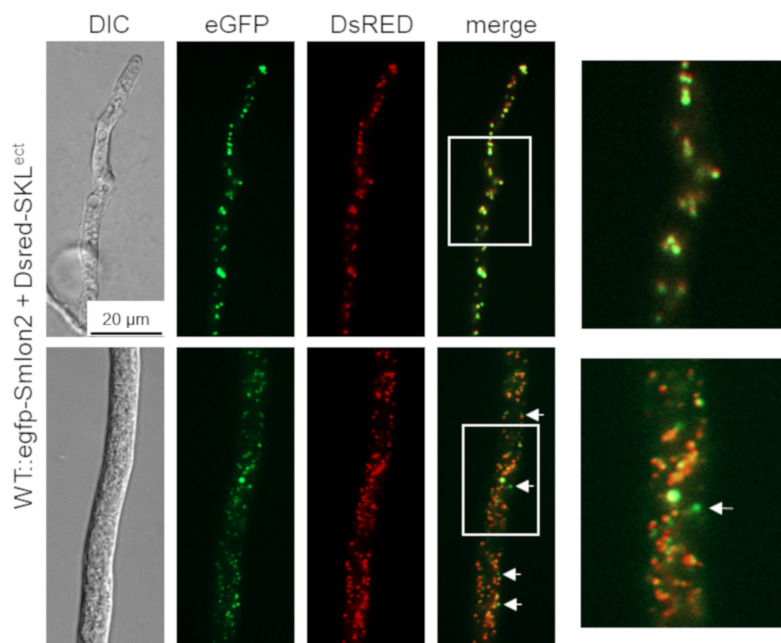
Co-localization of SmLON2 and the glyoxysomal reporter protein DsRED-SKL. Fluorescence microscopy was performed with the *S. macrospora* WT strain expressing eGFP-SmLON2 and the glyoxysomal reporter protein DsRED-SKL (WT::egfp-Smlon2 + Dsred-SKL^ect^). The upper panel shows a hyphal tip and the lower panel a subapical hyphal compartment. Arrows point to SmLON2 structures not co-localizing with glyoxysomes. Scale bar is indicated. DIC, differential interference contrast. Detail 4-fold enlargements of the merge picture are indicated with a frame and shown at the right margin.

To determine the cellular localization of SmLON2, we performed fluorescence microscopy of N-terminally tagged eGFP-SmLON2. The tagged protein localized to distinct spots varying in size, which presumably represent glyoxysomes (Figure 2). To verify the localization of SmLON2 in glyoxysomes, we performed co-transformation of the WT strain with pegfp-Smlon2 expressing the genomic version of Smlon2 under control of the endogenous promoter fused to *egfp* and pDsred-SKL encoding the red glyoxysomal marker protein DsRED-SKL with a PTS1 sequence [32]. The merged fluorescence images mostly revealed co-localization of DsRED-SKL and eGFP-SmLON2 in glyoxysomes of hyphal tips and subapical hyphal compartments. However, in some instances, the punctate accumulations of SmLON2 in the cytoplasm seemed to not co-localize with glyoxysomal structures (Figure 2).

**Figure 3:**
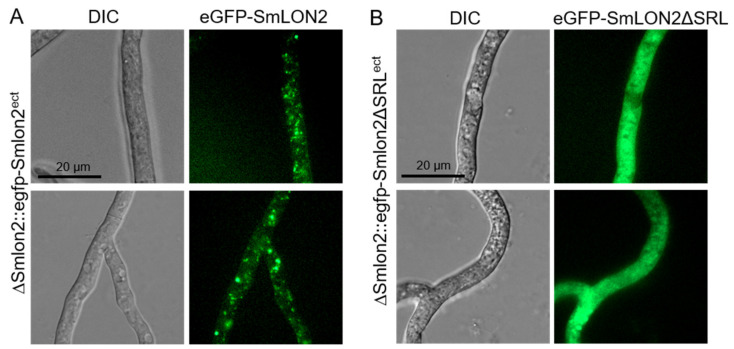
Localization of SmLON2 and SmLON2ΔSRL tagged with eGFP. (**A**) Fluorescence microscopy was performed with the deletion mutant ΔSmlon2 carrying plasmid pegfp-Smlon2 and (**B**) plasmid pegfp-Smlon2ΔSRL, respectively. Scale bars are indicated. DIC, differential interference contrast.

To analyse localization in a deletion mutant, a ΔSmlon2 deletion strain was created by homologous recombination of a *hph* deletion cassette flanked by upstream and downstream sequences of *Smlon2*. Deletion of *Smlon2* was confirmed by PCR and Southern blot (Figure S2). To determine the cellular localization of SmLON2, we performed fluorescence microscopy of N-terminally tagged eGFP-SmLON2 and a variant of the fusion protein, GFP-SmLON2ΔSRL, lacking the C-terminal PTS1 SRL. Both, the WT and the mutated version of *Smlon2* lacking the PTS1 were expressed in ΔSmlon2 deletion mutant (Figure 3).

eGFP-SmLON2 localized to distinct spots varying in size, which represent glyoxysomes (Figure 3A). The same localization was observed when SmLON2 was N-terminally fused to TagRFP-T instead of eGFP (Figure S3). In contrast, the eGFP tagged mutant version SmLON2ΔSRL was found evenly distributed within the cytoplasm (Figure 3B). This resembles the localization of free eGFP (Figure S4). 

### 3.3. Deletion of Smlon2 Leads to Defects in Fruiting-Body Development and Ascospore Production

**Figure 4:**
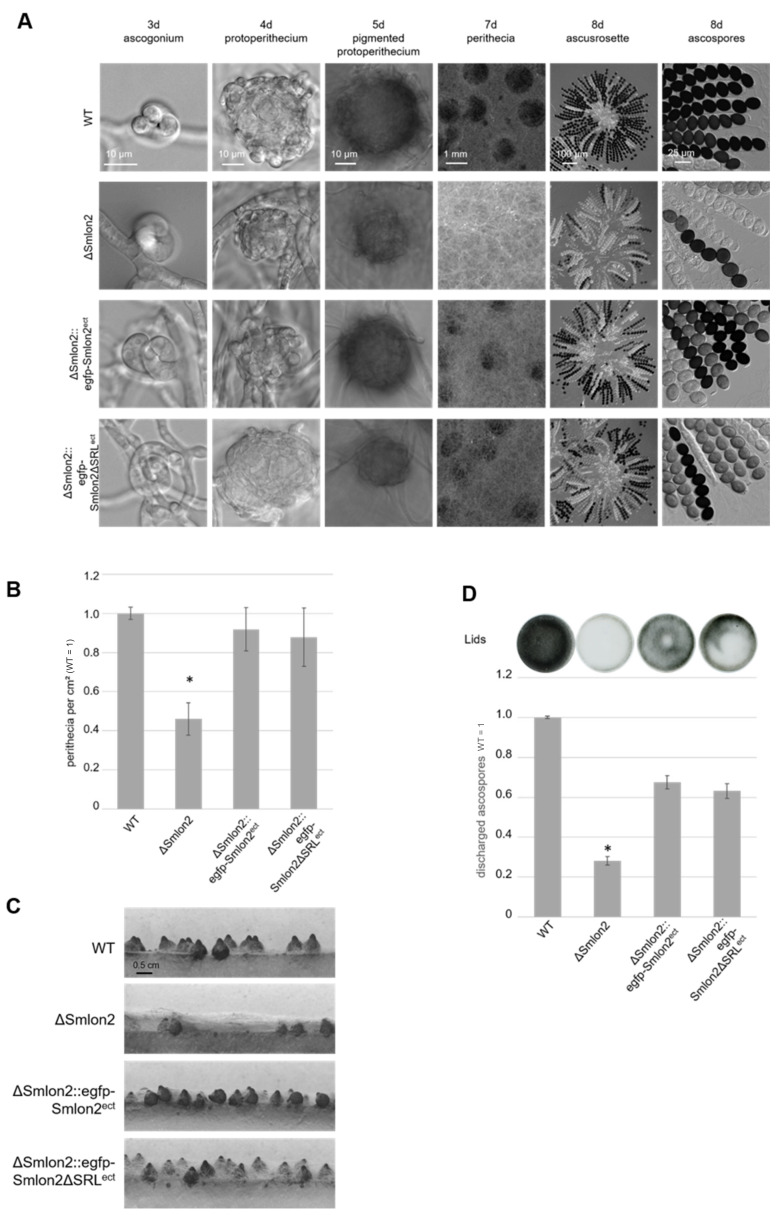
Phenotypic analysis of *S. macrospora* WT, ΔSmlon2 mutant strain and complementation strains expressing variants of *Smlon2*. Microscopic investigation of sexual development of WT compared to ΔSmlon2 and the complementation strains ΔSmlon2::egfp-Smlon2^ect^ and ΔSmlon2::egfp-Smlon2ΔSRL^ect^. (**A**) Strains were grown on slides covered with fructification medium (SWG) at 27 °C, and photographs were taken at indicated days. (**B**) Perithecia were counted per cm^2^ after 8 d. Number of perithecia shown are averages relative to the WT from ten technical replicates of three independent experiments (*n* = 30). (**C**) Side view of the strains after inoculation on solid SWG medium for 10 d at 27 °C. The solid medium was sliced into thin strips that were microscopically investigated. (**D**) Ejected ascospores, after 10 d of growth, from the lid of a petri dish (56.7 cm^2^) for the different strains. The results are averages relative to the WT from 5 technical replicates of three independent experiments (*n* = 15). A representative picture of a lid is shown above the diagram. (*) Asterisks indicate significant differences to the WT of *p* < 0.0001 according to Student’s t-test. Scale bars are shown in the images.

To examine the role of the putative protease SmLON2 during vegetative growth and sexual development in comparison to the WT strain, the vegetative growth rate of the ΔSmlon2 deletion was determined. The growth rate of the deletion mutant did not change under normal conditions (SWG medium) (Figure S5). However, the ΔSmlon2 strain exhibited defects in sexual development when compared to the WT strain, and the complementation strain ectopically expressing the genomic version of *Smlon2* under control of the endogenous promoter fused to *egfp* (ΔSmlon2::egfp-Smlon2^ect^) and a variant of the same *egfp*-*Smlon2* construct encoding a protein without the C-terminal PTS1 (ΔSmlon2::egfp-Smlon2ΔSRL^ect^) (Figure 4A).

All strains completed their life cycles and produced ascospores within eight days. In *S. macrospora*, the life cycle starts with the germinating ascospores that develop a haploid vegetative mycelium. In all strains, female gametangia (ascogonia) and spherical, unpigmented fruiting-body precursors (protoperithecia) could be observed after three and four days, respectively. After five days, these developed into melanin-pigmented large protoperithecia. Then, self-fertilization events, karyogamy, meiosis and a postmeiotic mitosis occurred in the maturing perithecia leading to eight linearly ordered black ascospores per ascus (approximately 200 asci per perithecium; Figure 4A). In comparison to the WT, the deletion mutant ΔSmlon2 produced a significantly reduced number of perithecia per cm^2^. This defect was complemented by both complementation constructs (Figure 4B,C). The loss of *Smlon2* enhanced the growth of aerial mycelium. The increase in aerial mycelium was not quantified, but could be clearly seen in Figure 4A,C. Due to the increase of aerial mycelium, perithecia were hard to identify in the plain view of the plates, but could be detected in the side view (Figure 4A,C). This change in morphology was only partially complemented by the ectopic copy of the WT *Smlon2* gene under control of its endogenous promoter and its variant *Smlon2ΔSRL* lacking PTS1 (Figure 4A,C). In WT, at day seven of development ascospores started to be forcibly ejected through a pore at the neck of the perithecium. Ascospore ejection lasts two to three days, leading to the blackening of the petri dish lid (Figure 4D). Ascus rosettes of the deletion mutant harbored an increased number of asci with only white immature ascospores. A quantitative analysis revealed that the number of discharged ascospores was significantly reduced in the ΔSmlon2 mutant in comparison to the WT strain (Figure 4D). This defect was partially complemented by ectopic copies of both *Smlon2* gene variants.

### 3.4. The Mutant ΔSmlon2 Exhibited an Increased Sensitivity against Oxidative and Nutrient Stresses

Recently, we reported that a *S. macrospora* mutant lacking the pexophagy receptor SmNBR1 was affected in vegetative growth and development under nutrient limiting and oxidative-stress conditions [14]. For this reason, we tested whether absence of the glyoxysomal protease SmLON2 leads to growth and developmental defects under different stress conditions.

**Figure 5:**
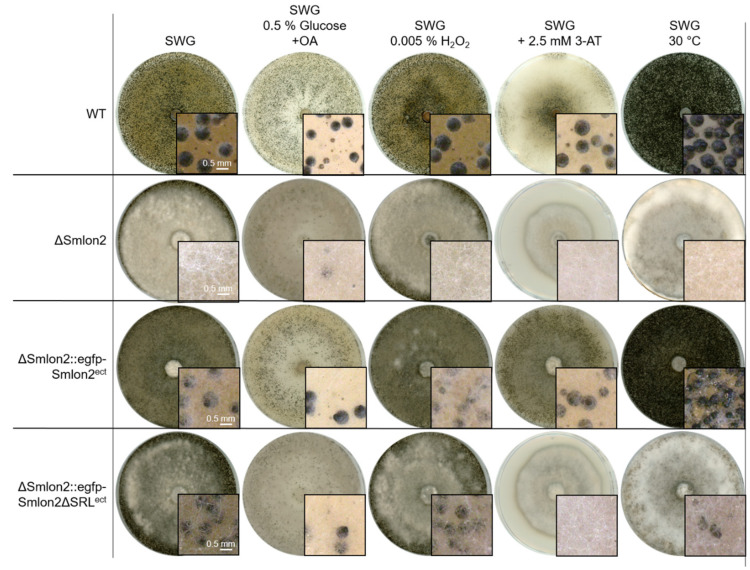
Sexual development of *S. macrospora* WT, ΔSmlon2 and the complementation strains expressing variants of *Smlon2* under different stress conditions. WT, ΔSmlon2 and complementation strains expressing the *S. macrospora* (ΔSmlon2::egfp-Smlon2^ect^) and the mutated version of *Smlon2* (ΔSmlon2::egfp-Smlon2ΔSRL^ect^), respectively, were grown under normal conditions on fructification medium (SWG) at 27 °C. For the induction of β-oxidation in microbodies the amount of glucose in the SWG medium was reduced to 0.5% and 0.15% oleic acid (OA) was added. Oxidative stress was induced by the addition of 0.005% H_2_O_2_ and amino-acid starvation was induced by adding 3-amino-1,2,4-triazole (3-AT, 2.5 mM) to SWG medium. Temperature stress was performed by cultivation of the strains at 30 °C. Pictures of agar plates were taken after 8 d. Magnifications are shown in the image details. Scale bars are indicated.

First, we tested both vegetative growth and sexual development of WT, ΔSmlon2 and the complementation strains, either on medium containing 0.15% oleic acid (OA) as carbon source or by inducing oxidative stress by adding 0.005% H_2_O_2_ to the growth medium (Figure 5). On both media, all strains were able to form perithecia. However, in the mutant and the complementation strain carrying the *Smlon2ΔSRL* version, production of aerial mycelium was increased (Figure 5). When increasing the concentration of added H_2_O_2_ to 0.01%, the deletion mutant ΔSmlon2 could not grow at all, while the WT strain and complementation strains were able to grow on media containing up to 0.02% H_2_O_2_ (Figures S5 and S6).

Starvation conditions were induced by adding the drug 3-amino-1,2,4-triazole (3-AT), resulting in histidine starvation. Under this starvation condition, WT and the complementation strain carrying the WT *Smlon2* gene displayed normal sexual development and vegetative growth, whereas ΔSmlon2 and the complementation strain carrying *Smlon2ΔSRL* were not able to form perithecia (Figure 5). In addition, the vegetative growth rate of the mutant strain was significantly reduced under this condition (Figure S5). Heat stress, provoked by cultivation at 30 °C, led to an increased production of perithecia in WT and the complementation strain carrying the *egfp*-tagged WT *Smlon2* gene, but to a reduction of perithecia in the deletion strain ΔSmlon2 and the complementation strain ΔSmlon2::egfp-Smlon2ΔSRL (Figure 5). In contrast, the vegetative growth rate was not impaired in any of the strains under heat stress condition (Figure S5).

These results showed that fatty-acid metabolism, oxidative and nutrient stress resistance were impaired in the ΔSmlon2 mutant.

### 3.5. Starvation Stress Resulted in an Enhanced Vacuolar Degradation of Glyoxysomes in the ΔSmlon2 Mutant

In *A. thaliana,* dysfunctional peroxisomes of *lon2* mutants were shown to be increasingly degraded by pexophagy [50,51]. In order to examine whether the lack of SmLON2 led to an enhanced degradation of glyoxysomes in *S. macrospora*, we expressed the glyoxysomal marker protein DsRED-SKL in the WT strain and ΔSmlon2 deletion mutant and compared the distribution of glyoxysomes in both strains under normal and starvation stress conditions.

**Figure 6:**
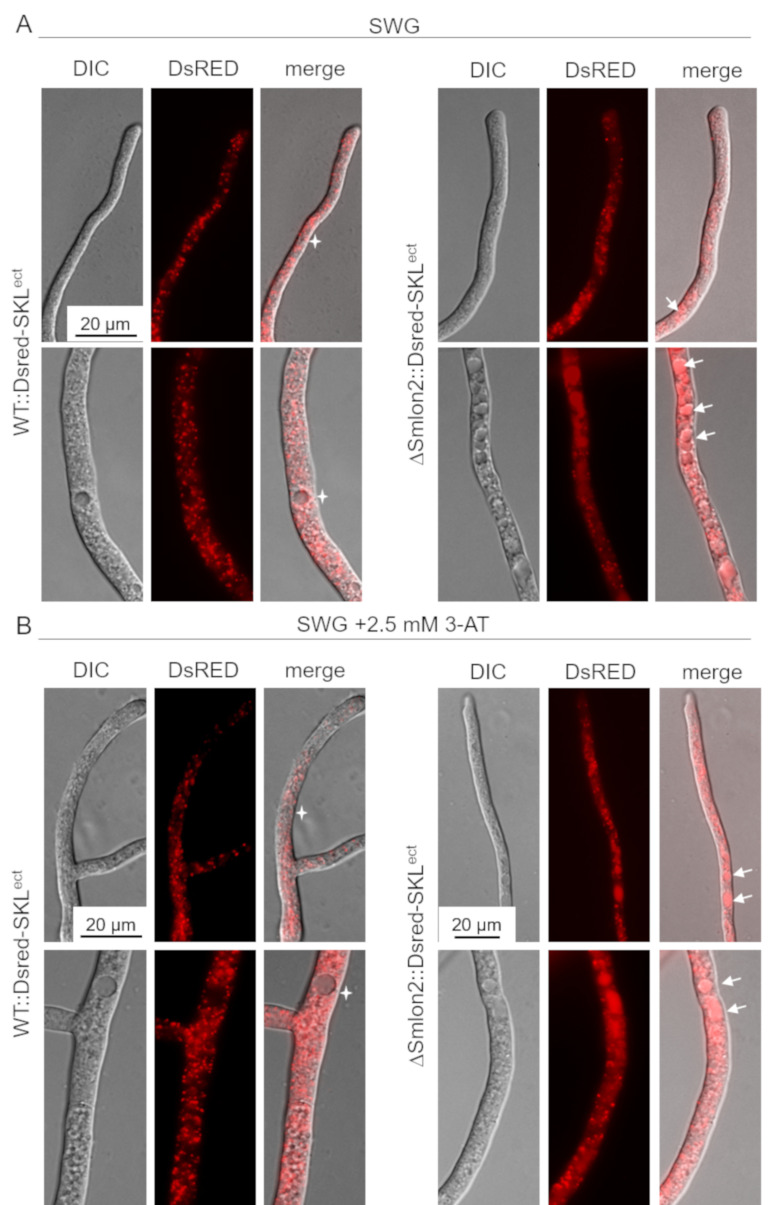
Localization studies of the glyoxysomal marker protein DsRED-SKL in WT and ΔSmlon2. Fluorescence microscopy was performed with WT and deletion mutant ΔSmlon2 expressing DsRED-SKL. **Upper panel**: hyphal tip; **lower panel**: subapical hypha. (**A**) Growth of hyphae under normal growth conditions on SWG medium. (**B**) Induction of nutrient starvation by supplementing SWG with 2.5 mM 3-AT. DIC, differential interference contrast. Vacuoles with DsRED fluorescence are indicated by arrows, vacuoles without DsRED fluorescence are marked stars. Scale bars are indicated.

In the WT strains, DsRED-SKL localized to punctate glyoxysomal structures and was excluded from vacuoles, in both young hyphal tips and older subapical hyphae. However, in the ΔSmlon2 mutant strain, DsRED-SKL fluorescence of small vacuoles in hyphal tips and larger vacuoles in older hyphal compartments was enhanced. In addition, the number of vacuoles was increased in the older subapical hyphae of the deletion mutant (Figure 6A). Under nutrient starvation conditions SWG + 2.5 mM 3-AT), the vacuolar localization of DsRED-SKL was similarly increased in the ΔSmlon2 mutant. Furthermore, proliferation and enlargement of vacuoles could be observed in hyphal tips and subapical cells of the deletion mutant under nutrient starvation conditions (Figure 6B). Together, these data suggest that the lack of protease SmLON2 lead to an increase of DsRED-SKL fluorescence in vacuoles under normal conditions and starvation conditions.

## 4. Discussion

Here, we describe the role of a glyoxysomal Lon protease in vegetative growth and sexual development of *S. macrospora*. Most fungi, with exception of the yeasts *S. cerevisiae* and *Candida glabrata*, possess two Lon isoforms localized either to mitochondria or peroxisomes [23,24,25,52,53]. Similarly, we identified two Lon proteases predicted to localize in mitochondria and microbodies, respectively, in *S. macrospora*. Both proteins are highly homologous to the mitochondrial and peroxisomal Lon protease from other eukaryotes with a typical Lon domain at the N-terminal region, an ATPase domain in the central part and a protease domain at the C-terminal region (Figure 1).

Deletion of the peroxisomal *lon* gene in *H. polymorpha*, *Penicillium chrysogenum* and *Thermomcyces lanuginosus* resulted in enhanced oxidative stress levels and vegetative growth defects under specific conditions [23,24,25]. However, the role of a glyoxysomal protein homeostasis in sexual development has not been analyzed yet. Deletion of *Smlon2* in *S. macrospora* resulted in a decreased production of perithecia and ascospores (Figure 4). A similar phenotype has been observed in the *S. macrospora* mutant lacking the pexophagy receptor SmNBR1. Like in the ΔSmnbr1 mutant, a reduced number of perithecia were embedded in a compact aerial mycelium and the number of discharged ascospores was significantly lower in the ΔSmlon2 mutant than in the WT [14]. This result suggests that similar to hampered pexophagy, a disturbed glyoxysomal protein homeostasis impairs sexual development of *S. macrospora*. In contrast to the reduced number of sexual spores produced by the *S. macrospora* ΔSmlon2 mutant, the production of asexual conidia increased in a *T. lanuginosus* mutant lacking the peroxisomal PLon [25]. In the close relative of *S. macrospora*, *P. anserina,* peroxisomes were shown to perform different functions during ascus development and ascospore formation. Morphology and dynamics of peroxisomes are different in vegetative cells present within perithecia and change morphologically in sexual cells and during the sexual cycle [54,55,56]. Therefore, the impact of the disturbed glyoxysomal protein homeostasis possibly differs in vegetative and sexual cells. 

Different stress conditions (increased fatty-acid metabolism, oxidative stress, nutrient stress and heat stress) enhanced defects in sexual development of the ΔSmlon2 mutant. Unlike the ΔPLon mutant of *T. lanuginosus* but similar to the *P. chrysogenum pln* mutant, ΔSmlon2 displayed a normal growth rate on fructification medium (Figure S5) [24,25]. However, in comparison to the WT, the mutant exhibited an increased formation of aerial mycelium. Furthermore, we observed no significant impairment of the vegetative growth under heat stress or on medium containing increased amounts of oleic acid, indicating that oleic acid was not toxic for the ΔSmlon2 mutant as previously described for the *P. chrysogenum pln* mutant [24]. However, when exposed to exogenous peroxide, the vegetative growth rate of the ΔSmlon2 was significantly reduced or completely abolished at higher H_2_O_2_ concentrations. Moreover, oxidative stress resistance is impaired in the ΔSmlon2 mutant may be due to the accumulation of oxidized glyoxysomal matrix proteins and cytoplasmic proteins (Figures S5 and S6).

Like in *P. chrysogenum*, the absence of SmLON2 seemed not to interfere with the import of matrix proteins into glyoxysomes, since the import of DsRED-SKL into glyoxysomes was similar in the ΔSmlon2 mutant and the WT (Figure 6) [24]. Furthermore, no increase in the number or the size of peroxisomes could be observed in the ΔSmlon2 mutant, as was previously described for *H. polymorpha* and *A. thaliana,* respectively [23,51]. The major difference between the ΔSmlon2 mutant and WT is that already in young hyphal tips, the DsRED-SKL fluorescence was enhanced in vacuoles, in particular under nutrient stress conditions (Figure 6). This effect might occur due to an increased pexophagy of dysfunctional glyoxysomes accumulating non-functional and improper folded proteins. Alternatively, the enhanced vacuolar fluorescence might be a consequence of enhanced autophagy of cytosolic DsRED-SKL, because protein import into glyoxysomes might be disturbed.

Interestingly, SmLON2ΔSRL lacking PTS1 partially complemented defects of the ΔSmlon2 mutant in perithecia and ascospore formation (Figure 4). Additionally, SmLON2ΔSRL complemented increased sensitivity to oxidative stress conditions (Figures S5 and S6). This suggests an additional function of SmLON2 in the cytosol. 

Localization studies clearly revealed transport of eGFP and TagRFP-T-tagged SmLON2 into glyoxysomes (Figure 2 and Figure S3). In addition, the glyoxysomal marker DsRED-SKL predominantly co-localized with eGFP-SmLON2. However, in some cases, green EGFP-SmLON2 foci were observed in the cytoplasm that do not co-localize with DsRED-SKL (Figure 2), whereas the tagged SmLON2 variant without the PTS1 signal was clearly distributed in the cytoplasm (Figure 3). Therefore, SmLON2 might exist in two dually targeted isoforms, one localized to the cytoplasm and one to glyoxysomes. In recent years, dually targeted isoforms of peroxisomal proteins have been reported from several eukaryotes, including isoforms carrying out the same or different functions in microbodies and the cytoplasm. Therefore, different mechanisms mediating dual targeting of peroxisomal proteins have been described, among them the generation of different transcripts from one gene and inefficient transport of proteins into microbodies, due to inefficient or modified targeting signals [57]. 

Previously, it has been reported that posttranscriptional A-to-I RNA editing occurs during sexual development in *S. macrospora* resulting in nonsynonymous and synonymous codon changes, stop codon loss, and premature stop codon correction in pseudogenes [58,59,60]. We checked whether *Smlon2* is posttranslational modified by RNA editing and identified in the RNA editing database of *S. macrospora* (personal communication kindly provided by R. Lütkenhaus, I. Teichert and M. Nowrousian, Ruhr University Bochum, Germany) that *Smlon2* (*SMAC_00912*) contains six edited sites with four editing sites leading to nonsynonymous codon changes. RNA editing of the *Smlon2* transcript does not affect the PTS1 motive or the catalytic dyad of the protease domain. One site K245E is located in the N-terminal LON domain, one site N594D in the ATPase domain and two, I740M and S753G, in the protease domain (Figure S7). How these changes alter the function of the protein can currently not be answered. Nonetheless, RNA editing of *Smlon2* might alter protein properties, leading to dual targeting of the protein. 

In plants, two functions of LON2 were dissected. The chaperone function of LON2, but not its protease activity, suppresses autophagy. Furthermore, the protease function of LON2 was shown to interfere with its chaperone function, suggesting that intramolecular modulation between both functions of LON2 regulates degradation of peroxisomes by autophagy [51].

In conclusion, SmLON2 is involved in fruiting-body development, ascosporogenesis and stress resistance in *S. macrospora*. Our data suggest that SmLON2 might be a dually targeted protease having functions in glyoxysomes and in the cytoplasm. Which specific functions the two isoforms of SmLON2 have and whether RNA editing plays a role in fulfilling its functions remains so far elusive, but will be addressed in the future.

## Figures and Tables

**Table 1:** Overview of strains used and constructed in this study.

Strain	Genotype	Source
***Escherichia coli***		
MACH1	*ΔrecA1398, endA1, tonA, Φ80ΔlacM15, ΔlacX74, hsdR,* (rK-mK+)	Invitrogen
***Saccharomyces cerevisiae***		
PJ69-4A	*MATa, trp1-901, leu2-3,112, ura3-52, his3-200, ga14Δ, ga18OΔ LYS2::GALl-HIS3, GAL2-ADE2, met2::GAL7-lacZ*	[28]
***Sordaria macrospora***		
DSM997	Wild type (WT)	DSMZ
S23442	mutation in *fus1-1* gene, brownish ascospores	[34]
Δku70	Δku70::nat^R^	[36]
WT::1783-1^ect^	ectopic integration of p1783-1 into DSM997; *hyg^R^*, ssi, fertile, *Pgpd::egfp::TtrpC*	[15]
WT::RHN1^ect^	ectopic integration of pRHN1 into DSM997; *nat^R^*, ssi, fertile, *Pgpd::Dsred::TtrpC*	[14]
WT::TagRFP-T^ect^	ectopic integration of ptRFP_nat into DSM997; *nat^R^*, ssi, fertile, *Pccg1::Tagrfp-t::TtrpC*	This study
WT::Dsred-SKL^ect^	ectopic integration of pDsred-SKL into S48977; *nat^R^*, ssi, fertile, *Pgpd::Dsred-SKL::TtrpC*	[32]
ΔSmlon2	ΔSmlon2::hyg^R^, ssi	This study
WT::egfp-Smlon2^ect^+Dsred-SKL^ect^	ectopic integration of pDsred-SKL and pegfp-Smlon2 into DSM997; *nat^R^*, *hyg^R^* ssi, fertile, *Pgpd::Dsred-SKL::TtrpC; PSmlon2:: ORF egfp+Smlon2::TSmlon2*	This study
ΔSmlon2::egfp-Smlon2^ect^	ectopic integration of pegfp-Smlon2 into ΔSmlon2; *nat^R^*, *hyg^R^*, ssi, fertile, *PSmlon2:: ORF egfp+Smlon2::TSmlon2*	This study
ΔSmlon2::Tagrfp-t-Smlon2^ect^	ectopic integration of ptrfp-Smlon2 into ΔSmlon2; *nat^R^*, *hyg^R^*, ssi, fertile, *PSmlon2:: ORF trfp+Smlon2::TSmlon2*	This study
ΔSmlon2::egfp-Smlon2ΔSRLt^ect^	ectopic integration of pegfp-Smlon2ΔSRL into ΔSmlon2; *nat^R^*, *hyg^R^*, ssi, fertile, *PSmlon2:: ORF egfp+Smlon2 deletion of aa 935-937 (SRL)::TSmlon2*	This study
ΔSmlon2::Dsred-SKL^ect^	ectopic integration of pDsred-SKL into ΔSmlon2; *nat^R^*, *hyg^R^*, ssi, fertile, *Pgpd::Dsred-SKL::TtrpC*	This study

*nat*^R^: nourseothricin resistant, *hyg^R^*: hygromycin resistant; *aa*: amino acid, *ORF*: open reading frame, *Pccg1:* promoter of the *clock controlled gene 1* of *Neurospora crassa; Pgpd:* promoter of the glyceraldehyde-3-phosphate dehydrogenase gene of *Aspergillus nidulans; TtrpC:* terminator of the anthranilat synthase gene of *Aspergillus nidulans*; ssi: single spore isolate; SKL: peroxisomal targeting sequence Ser-Lys-Leu; SRL: peroxisomal targeting sequence Ser-Arg-Leu; *Dsred*: gene for red fluorescence protein (DsRED) of *Discosoma* species; *egfp*: gene for green fluorescence protein enhanced green fluorescent protein (eGFP) of *Aequorea victoria; trfp*: gene for red fluorescence protein TagRFP-T of *Entacmaea quadricolor*.

## Data Availability

Not applicable.

## Supplementary Materials

The following are available online at https://www.mdpi.com/2309-608X/7/2/82/s1, Table S1: List of plasmids used in this study, Table S2: List of primers used in this study, Figure S1: Phylogenetic tree of Lon proteases from fungi, plants, animals and bacteria, Figure S2: Verification of the deletion of *Smlon2* in mutant ΔSmlon2, Figure S3: Localization of the Lon protease SmLON2 fused with tRFP, Figure S4: Localization of free eGFP, tRFP and DsRED, Figure S5: Vegetative growth of WT, ΔSmlon2 and the complementation strains expressing variants of *Smlon2* under different stress conditions, Figure S6: Sexual development of *S. macrospora* WT, ΔSmlon2 and the complementation strains expressing variants of *Smlon2* under oxidative stress conditions, Figure S7: RNA A-I editing of *Smlon2*.
