# Supplementary material for: The Glyoxysomal Protease LON2 Is Involved in Fruiting-Body Development, Ascosporogenesis and Stress Resistance in Sordaria macrospora
Source: J Fungi (Basel). 2021 Jan 26;7(2):82. doi: 10.3390/jof7020082 (PMC7911957; doi:10.3390/jof7020082)
Supplement: Supplementary file 1 [file jof-07-00082-s001.pdf]

Supplementary material

Table S1: List of plasmids used in this study.

Plasmid	Characteristics	Source
pHAN1	*amp*^{R}*, his-3, Pccg1::ha*	[1]
pRS426	*amp*^{R}*, ura3*	[2]
pRS-nat	*amp*^{R}*, ura3, nat*^{R}	[3]
pRS-hyg	*amp*^{R}*, ura3, hyg*^{R}	[4]
p1783-1	*amp*^{R}*, ura3, hyg*^{R,} *Pgpd::egfp::TtrpC*	[5]
pRHN1	*amp*^{R}*, ura3, nat*^{R} *Pgpd::Dsred::Ttrpc*	[6]
pDsred-SKL	*amp*^{R}*, nat*^{R} *Pgpd::Dsred-SKL::TtrpC*	[7]
pTagRFP-T_nat	*amp*^{R}*, nat*^{R} *Pccg1::TagRFP-T::TtrpC*	This study
pAL5Lifeact	*amp*^{R}*, bar*^{R} *Pccg1::tRFP::TtrpC*	[8]
pegfp-Smlon2	*amp*^{R}*, ura3, nat*^{R} *PSmlon2::ORF egfp+Smlon2::TSmlon2*	This study
pegfp-Smlon2	*amp*^{R}*, ura3, hyg*^{R} *PSmlon2::ORF egfp+Smlon2::TSmlon2*	This study
ptrfp-Smlon2	*amp*^{R}*, ura3, nat*^{R} *PSmlon2::ORF trfp+Smlon2::TSmlon2*	This study

pegfp-Smlon2ΔSRL	*amp*^{R}*, ura3, nat*^{R}<br><br>*PSmlon2::ORF egfp+Smlon2 deletion of aa 935-937 (SRL)::TSmlon2*	This study
pSmlon2-KO	*amp*^{R}*, ura3,* 5'-flanking region and 3'-flanking region of *Smlon2* interrupted by the *hph*-cassette in pRS426	This study

nat^R: nourseothricin resistant, *hyg*^{R}: hygromycin resistant; *amp*^{R} ampicillin resistance; *bar*^{R}, Basta-resistance (bar) gene *ura3,* Orotidine-5'-phosphate decarboxylase gene of *S. cerevisiae; hph*, hygromycin B phosphotransferase gene, *Pgpd:* promoter of the glycerinaldehyd-3-phosphat-dehydrogenase-gene of *Aspergillus nidulans; TtrpC:* terminator of the anthranilat synthase gene of *Aspergillus nidulans*; SKL: peroxisomal targeting sequence Ser-Lys-Leu; SRL: peroxisomal targeting sequence Ser-Arg-Leu; *Dsred*: gene for red fluorescence protein (DsRED) of *Discosoma* species; *egfp*: gene for green fluorescence protein enhanced green fluorescent protein (eGFP) of *Aequorea Victoria*, *trfp*: gene for red fluorescence protein TagRFP-T of *Entacmaea quadricolor*.

Table S2: List of primers used in this study.

Name	Sequence (5‘-> 3‘)
pRSccg1	***GTAACGCCAGGGTTTTCCCAGTCACGACG*** TAGAAGGAGCAGTCCATCTG
Pccg1_RFP	***TTAATCAGCTCTTCGCCCTTAGACACCAT*** TTTGGTTGATGTGAGGGGTT
RFP-f	ATGGTGTCTAAGGGCGAAGAG
RFP-r-trpC	***TTTGATGATTTCAGTAACGTTAAGTGGAT*** TTACTTGTACAGCTCGTCCATGC
TrpC_F	GATCCACTTAACGTTACTGAAATCATCAAA
pRS426GFPrev	***GCGGATAACAATTTCACACAGGAAACAGC*** TCGAGTGGAGATGTGGAGTG
lon2-ko-5f	***GTAACGCCAGGGTTTTCCCAGTCACGACG*** GCATTCTCAGTCATCATTAG
lon2-ko-3r	***GCGGATAACAATTTCACACAGGAAACAGC*** CGTCTGGCTACCTACCTTAC
lon2-ko-5r-hph	***CCAAAAATGCTCCTTCAATATCAGTTAAC*** GGGGACGTCGGTCTATAGTG
lon2-ko-3f-hph	***GAGTAGATGCCGACCGGGAACCAGTTAAC*** ATTGGCTACCTCAAGGTAGT
hph-f	GTTAACTGATATTGAAGGAGCATTTTTGG
hph-r	GTTAACTGGTTCCCGGTCGGCATCTACTC
lon2-ko-v5f	GCTAGCGGGTCTAGATGTTGA
lon2-ko-v3r	GTGTAGCCAGTCGAGTCTGCA
tC1_0	CCTGGACGACTAAACCAAAA
h3_0	GATGGCTGTGTAGAAGTACT
lon2-s3-f	TCAGCGGCTATGCTCGAAGTC

lon2-s4-r	GATGCCTCCGACGGCCGTGAT
lon2-p-3r	***GTGAACAGCTCCTCGCCCTTGCTCACCAT*** GGGGACGTCGGTCTATAGTG
Smlon2P_trfp	***TTAATCAGCTCTTCGCCCTTAGACACCAT*** GGGGACGTCGGTCTATAGTG
GFP-f	ATGGTGAGCAAGGGCGAGGAGC
egfp-r-Smlon2	***ATCGTGACCGTCGGAGCTCGCACGGGAGC*** CTTGTACAGCTCGTCCATGC
trfp-r_Smlon2	ATGGTGTCTAAGGGCGAAGAG
Smlon2-f-ATG	GCTCCCGTGCGAGCTCCGACG
lon2-SRLr2	TCATTCGACGCTCGGGTAATCGTGTTCGCTGGGCC
lon2-SRLf	***GCGAACACGATTACCCGAGCGTCGAATGA*** ATTGGCTACCTCAAGGTAGT

Bold italics = overhangs

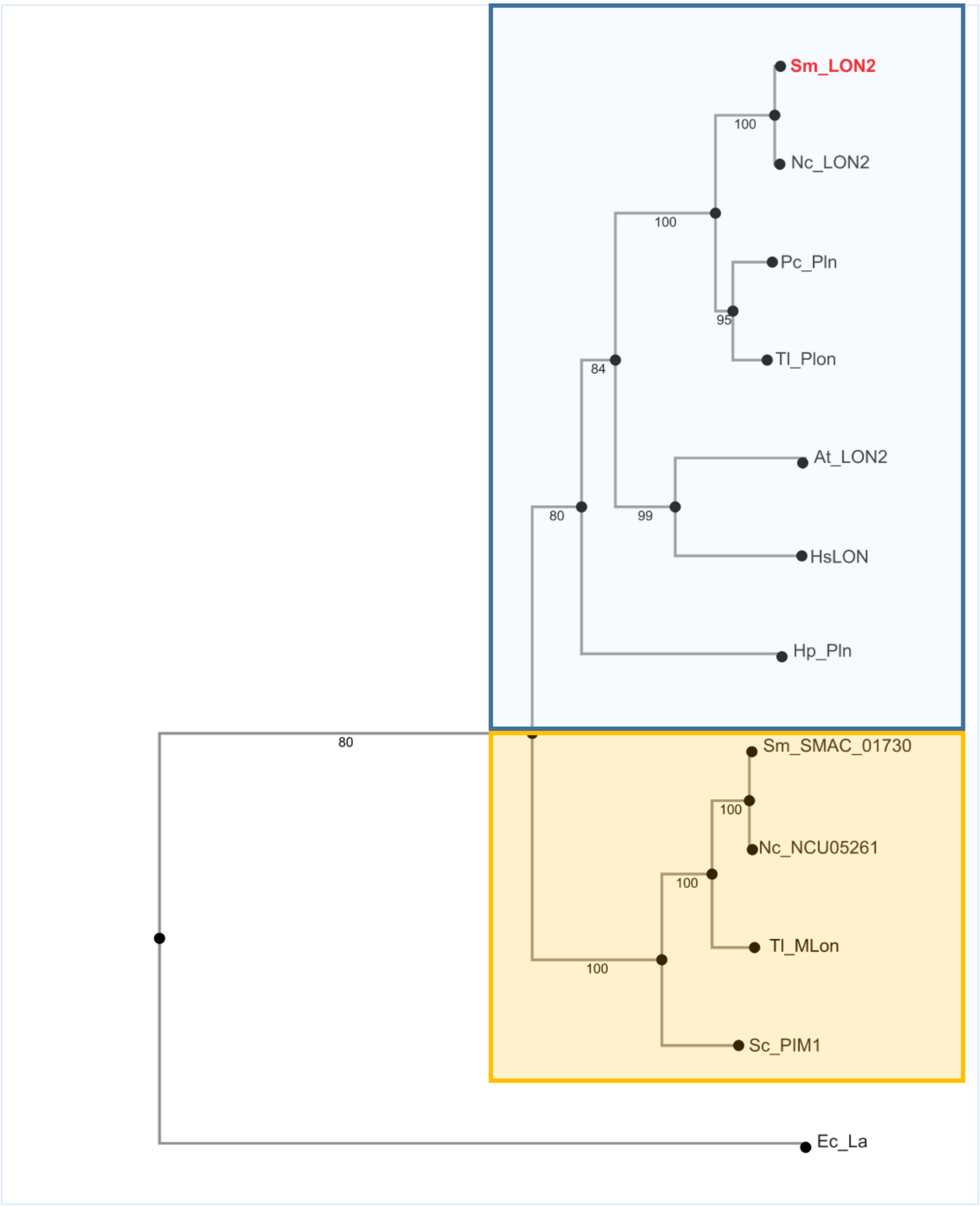

Figure S1: Phylogenetic tree of Lon proteases from fungi, plants, animals and bacteria.

The phylogenetic tree of Lon proteases was generated with the Neighbor Joining method. Orthologs were identified with BLASTP search using amino acid sequences of the *Sordaria*

macrospora SmLON2 (SMAC_00912) marked in red. The multiple sequence alignment and phylogenetic analysis was performed with MAFFT version 7 [9] using the amino acid sequence: Sm_LON2, *Sordaria macrospora* (XM_003349975.1); Sm_SMAC_01730, *S. macrospora* (KAA8635865.1); Nc_LON2, *Neurospora crassa* (XP_962516.1); Nc_NCU05261, *N. crassa* (XP_961826.1); Pc_Pln, *Penicillium chrysogenum*, (KZN88437.1); Tl_Plon, *Thermomyces lanuginosus* (Thela2p4_005149, https://gb.fungalgenomics.ca); Tl_Mlon, *T. lanuginosus* (Thela2p4_006664, https://gb.fungalgenomics.ca); Hp_Pln, *Hansenula polymorpha,* (ABB88892.1); At_LON2, *Arabidopsis thaliana* (NP_568675.1), Hs_LON, *Homo sapiens* (NP_113678.2); Sc_PIM1, *Saccharomyces cerevisiae* (P36775); Ec_La, *Escherichia coli* (CAD6055224.1). The bootstrap values based on 1000 replications are rounded to whole numbers and are indicated at the nodes. Lon-protease targeted to peroxisomes are framed in blue and proteins targeted to mitochondria are framed in orange.

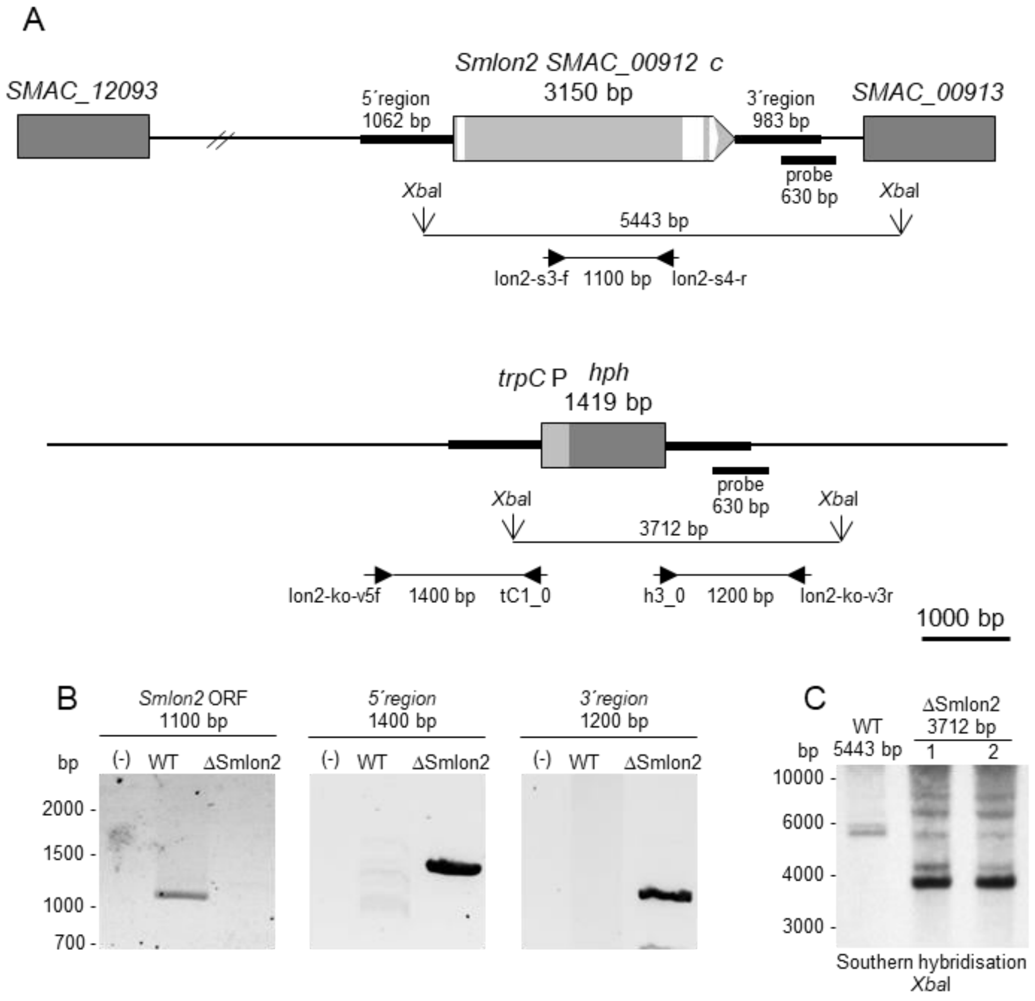

Figure S2: Verification of the deletion of *Smlon2* in mutant ΔSmlon2.

(A) Schematic representation of the *Smlon2* ORF and the 5' and 3' flanking region. After homologous recombination, the *hph* cassette replaced the entire *Smlon2* ORF. Primer combinations for PCR verification of the deletion, the corresponding sizes of fragments, the position of the restriction sites for *Xba*I and the probe for the Southern blot are indicated. (B) PCR verification of homologous integration of the *hph* cassette into the *Smlon2* locus. Genomic DNA was isolated from WT and ΔSmlon2 and tested with given primer combinations. Water serves as negative control (-). DNA-Ruler 1kb Plus. (C) Confirmation of the deletion by Southern blot. The isolated genomic DNA was hydrolyzed with the enzyme *Xba*I.

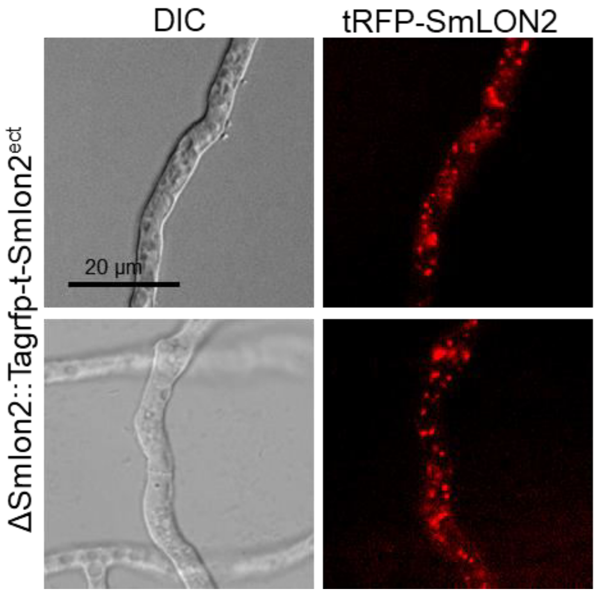

Figure S3: Localization of the Lon protease SmLON2 fused with tRFP. Fluorescence microscopic analysis of the ΔSmlon2 strain carrying plasmid ptrfp-Smlon2. DIC, differential interference contrast. Scale bar as indicated.

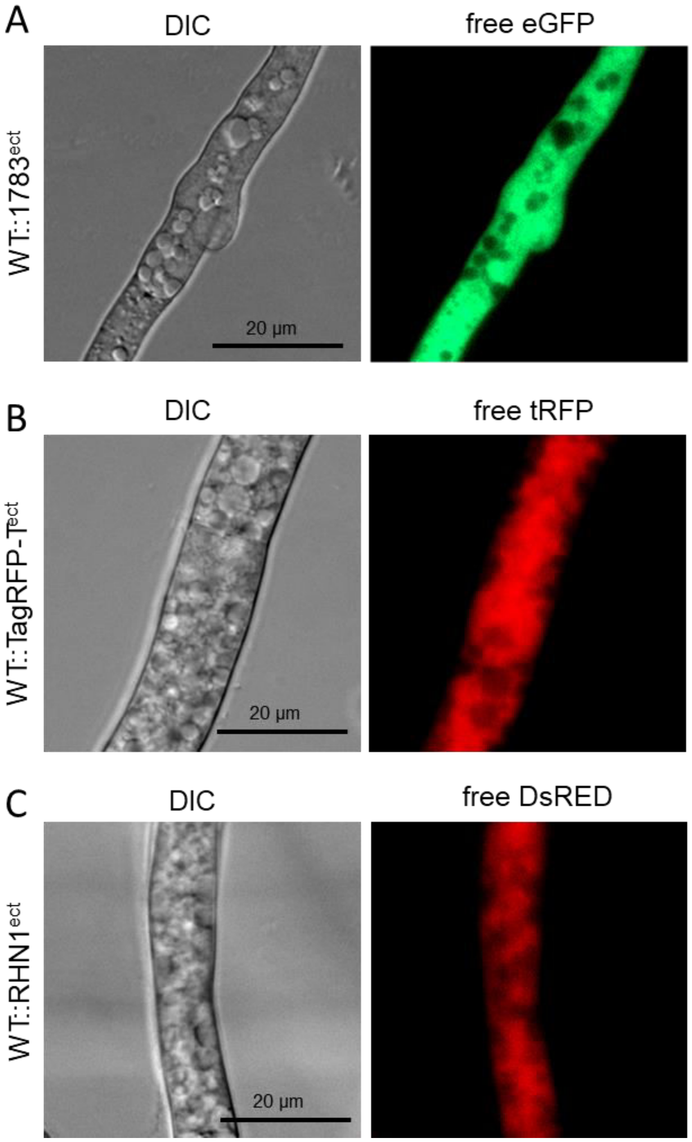

Figure S4: Localization of free eGFP, TagRFP-T and DsRED. Fluorescence microscopic analysis of the WT strain carrying plasmid p1783-1 (*egfp* under control of the *A. nidulans gpd* promoter; [5]), pTagRFP-T_nat (*trfp* under control the *Neurospora crassa ccg1* promoter) and pRHN1 (*Dsred* under control of the *A. nidulans gpd* promoter; [6]), respectively. DIC, differential interference contrast. Scale bars as indicated.

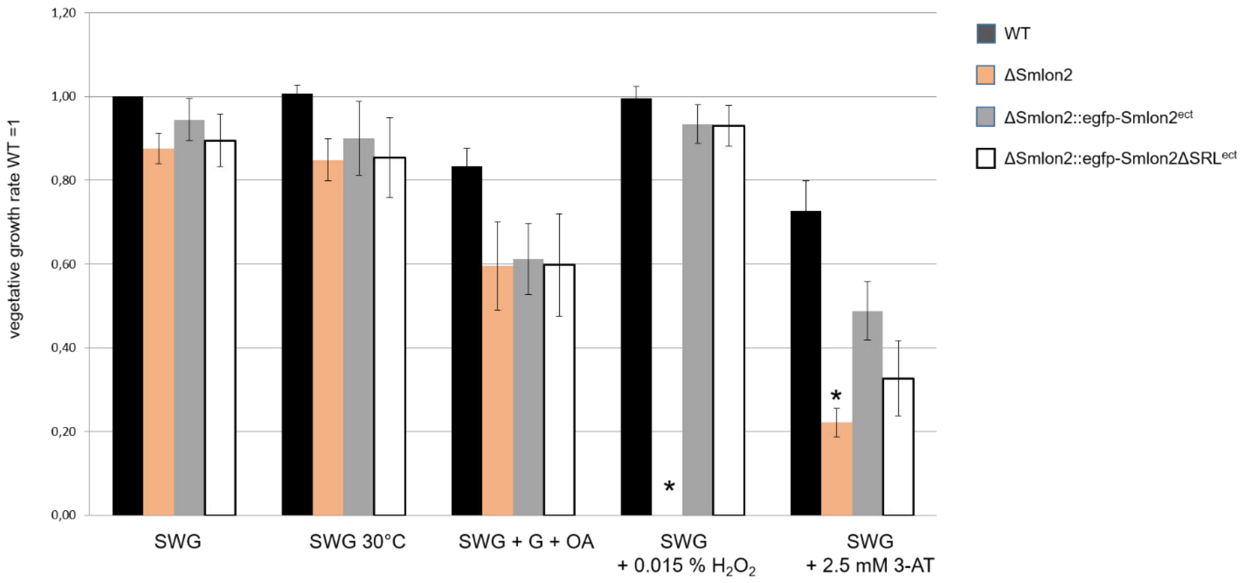

Figure S5: Vegetative growth of WT, ΔSmlon2 and the complemented strains expressing variants of *Smlon2* under different stress conditions. Growth rate of WT, ΔSmnbr1 and complemented strains expressing the *S. macrospora* WT *Smlon2* (ΔSmlon2::egfp-Smlon2^{ect}) and the mutated version of *Smlon2* (ΔSmlon2::egfp-Smlon2ΔSRL^{ect}) respectively, was determined in race tubes for five days. Normal conditions were on fructification medium (SWG) at 27°C, the growth of the WT was set to 1. Temperature stress was caused by cultivation of the strains at 30°C. For the induction of β-oxidation in microbodies we reduced the amount of glucose in the SWG medium to 0.5 % and added 0.15 % oleic acid (SWG + G + OA). Oxidative stress was induced by the addition of 0.015 % H_2O_2 (no growth of ΔSmlon2!) and amino-acid starvation was induced by adding 3-amino-1,2,4-triazole (SWG + 2.5 mM 3-AT) to the SWG medium. Data are means with standard deviations for three biological replicates and three independent experiments (n=15). Asterisks show significance difference to the WT analyzed by Students t-test ($p<0.0001$).

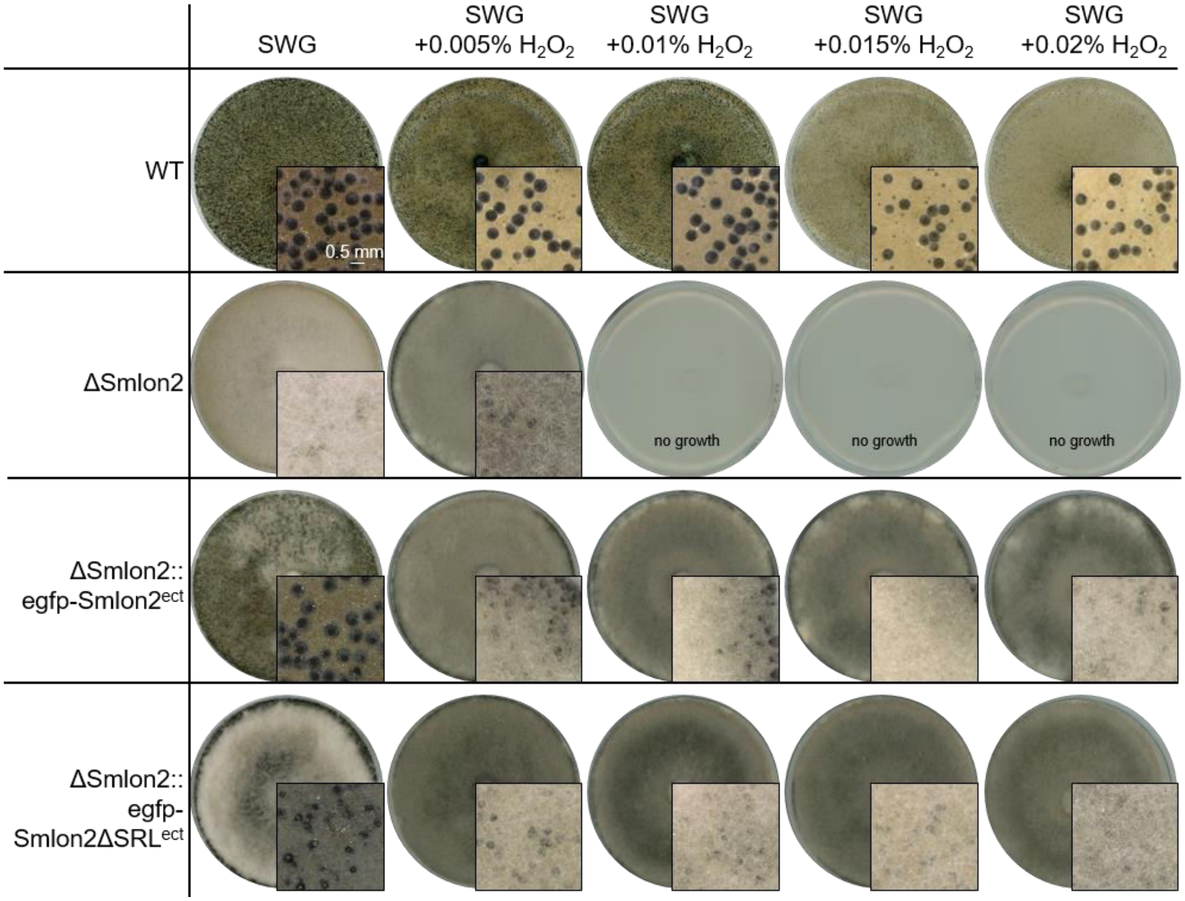

Figure S6: Sexual development of *S. macrospora* WT, ΔSmlon2 and the complemented strains expressing variants of *Smlon2* under oxidative stress conditions. WT, ΔSmnbr1 and complemented strains expressing the WT *Smlon2* gene (ΔSmlon2egfp::Smlon2^{ect}) and the mutated version of *Smlon2* (ΔSmlon2::egfp-Smlon2ΔSRL^{ect}) respectively, were grown under normal conditions on fructification medium (SWG) at 27°C. Oxidative stress was induced by increasing concentrations of H_2O_2 from 0.005 % to 0.02 %.

```
                10        20        30        40        50        60
LON2    MAPVRAPTVTIPLLPLPKGTILLPGVVQRIAVSSTRPDIASLLAAVYAKAASKTPNGRID
        ::::::::::::::::::::::::::::::::::::::::::::::::::::::::::::
LON2ed  MAPVRAPTVTIPLLPLPKGTILLPGVVQRIAVSSTRPDIASLLAAVYAKAASKTPNGRID
                10        20        30        40        50        60

                70        80        90       100       110       120
LON2    TIPIACVPLASPLLGPEGNLLIENGDNKNETSGDVDPAKATKADLFPYGVAAKITGVEGR
        ::::::::::::::::::::::::::::::::::::::::::::::::::::::::::::
LON2ed  TIPIACVPLASPLLGPEGNLLIENGDNKNETSGDVDPAKATKADLFPYGVAAKITGVEGR
                70        80        90       100       110       120

               130       140       150       160       170       180
LON2    GTGEFTLLVEGVTRIHVEKVIADKAYLEGKVSSYADPALITDSALEELFMSLKLLSRQFV
        ::::::::::::::::::::::::::::::::::::::::::::::::::::::::::::
LON2ed  GTGEFTLLVEGVTRIHVEKVIADKAYLEGKVSSYADPALITDSALEELFMSLKLLSRQFV
               130       140       150       160       170       180

               190       200       210       220       230       240
LON2    TILRLSSLLPQSSGTPGLSPLLARRLDFYIAKQKYPGALADFMANIVESTYEEKLQILTL
        ::::::::::::::::::::::::::::::::::::::::::::::::::::::::::::
LON2ed  TILRLSSLLPQSSGTPGLSPLLARRLDFYIAKQKYPGALADFMANIVESTYEEKLQILTL
               190       200       210       220       230       240

               250       260       270       280       290       300
LON2    IDVKERVAKVIELLDRQVTNIKNSMKITTITATSLPFPMDPDSTKPGKVKPPVKAPGQGV
        :::.::::::::::::::::::::::::::::::::::::::::::::::::::::::::
LON2ed  IDVEERVAKVIELLDRQVTNIKNSMKITTITATSLPFPMDPDSTKPGKVKPPVKAPGQGV
               250       260       270       280       290       300

               310       320       330       340       350       360
LON2    GMPFPPQGGFMGRGGNPDDEQEPNEIEELQKRLDAARLSPEAAKIADREIKRLKKIHPAQ
        ::::::::::::::::::::::::::::::::::::::::::::::::::::::::::::
LON2ed  GMPFPPQGGFMGRGGNPDDEQEPNEIEELQKRLDAARLSPEAAKIADREIKRLKKIHPAQ
               310       320       330       340       350       360

               370       380       390       400       410       420
LON2    AEYAVTRTYLETLAEIPWTATTDDRLGPDTLNRARKQLDDDHYGLDKVKKRLLEYLAVLR
        ::::::::::::::::::::::::::::::::::::::::::::::::::::::::::::
LON2ed  AEYAVTRTYLETLAEIPWTATTDDRLGPDTLNRARKQLDDDHYGLDKVKKRLLEYLAVLR
               370       380       390       400       410       420

               430       440       450       460       470       480
LON2    LKQAINDDVDIQIKQIEQELGVGSENGKEDAAQPAAVDLTVDEKVKAGGAKLEALKNRRM
        ::::::::::::::::::::::::::::::::::::::::::::::::::::::::::::
LON2ed  LKQAINDDVDIQIKQIEQELGVGSENGKEDAAQPAAVDLTVDEKVKAGGAKLEALKNRRM
               430       440       450       460       470       480

               490       500       510       520       530       540
LON2    VDKSPILLLVGPPGVGKTSLARSVATALGRKFHRISLGGVRDEAEIRGHRRTYVAAMPGL
        ::::::::::::::::::::::::::::::::::::::::::::::::::::::::::::
LON2ed  VDKSPILLLVGPPGVGKTSLARSVATALGRKFHRISLGGVRDEAEIRGHRRTYVAAMPGL
               490       500       510       520       530       540
```

```
              550       560       570       580       590       600
LON2   VVQGLKKVGVANPVFLLDEIDKVGGSSIHGDPSAAMLEVLDPEQNHNFTDHYVNIPIDLS
       :::::::::::::::::::::::::::::::::::::::::::::::::::::.::::::
LON2ed VVQGLKKVGVANPVFLLDEIDKVGGSSIHGDPSAAMLEVLDPEQNHNFTDHYVDIPIDLS
              550       560       570       580       590       600

              610       620       630       640       650       660
LON2   KVLFIATANSLDTIPAPLLDRMETIYIPGYTTLEKRHIAMQHLVPKQLRVNGLDESQVSF
       ::::::::::::::::::::::::::::::::::::::::::::::::::::::::::::
LON2ed KVLFIATANSLDTIPAPLLDRMETIYIPGYTTLEKRHIAMQHLVPKQLRVNGLDESQVSF
              610       620       630       640       650       660

              670       680       690       700       710       720
LON2   TPEVVSKIIESYTREAGVRNLEREISSVARGKAVEFADAKDSGHPENYNPQLTVDDLEKF
       ::::::::::::::::::::::::::::::::::::::::::::::::::::::::::::
LON2ed TPEVVSKIIESYTREAGVRNLEREISSVARGKAVEFADAKDSGHPENYNPQLTVDDLEKF
              670       680       690       700       710       720

              730       740       750       760       770       780
LON2   LGIEKFEEEIAEKTSRPGIVTGLVAYSSGGNGSILFIEVADMPGNGSVQLTGKLGDVLKE
       ::::::::::::::::::.:::::::::::::.:::::::::::::::::::::::::::
LON2ed LGIEKFEEEIAEKTSRPGMVTGLVAYSSGGNGGILFIEVADMPGNGSVQLTGKLGDVLKE
              730       740       750       760       770       780

              790       800       810       820       830       840
LON2   SVEVALTWVKAHAYELGLTQSPNENIMKDRSIHVHCPSGAVPKDGPSSGISQAIALISLF
       ::::::::::::::::::::::::::::::::::::::::::::::::::::::::::::
LON2ed SVEVALTWVKAHAYELGLTQSPNENIMKDRSIHVHCPSGAVPKDGPSSGISQAIALISLF
              790       800       810       820       830       840

              850       860       870       880       890       900
LON2   SGKAVPSTMAMTGEISLRGRITAVGGIKEKLIGALRAGVKTVLLPAQNRKDVKDLPQEVK
       ::::::::::::::::::::::::::::::::::::::::::::::::::::::::::::
LON2ed SGKAVPSTMAMTGEISLRGRITAVGGIKEKLIGALRAGVKTVLLPAQNRKDVKDLPQEVK
              850       860       870       880       890       900

              910       920       930
LON2   DGLEIIHVSHIWEAIRYVWPDGQWPSEHDYPSVESRL
       :::::::::::::::::::::::::::::::::::::
LON2ed DGLEIIHVSHIWEAIRYVWPDGQWPSEHDYPSVESRL
              910       920       930
```

Figure S7: RNA A-I editing of *Smlon2*. Alignment of the unedited (LON2) and edited (LON2ed) version of the SmLON2. Six sites were edited in the transcript of *Smlon2* (*SMAC-00912*): position 1379, 1807, 2429, 2866, 2906, and 3860. These lead to amino acid changes at positions K245E, N594D, I740M and S753G in the SmLON2 protein. Amino acid changes are

indicated in yellow, the LON domain is depicted in grey, the ATPase domain in blue and the protease domain in green.

References

1. Kawabata, T. and H. Inoue, *Detection of physical interactions by immunoprecipitation of FLAG- and HA-tagged proteins expressed at the his-3 locus in Neurospora crassa.* Fungal Genetics Newsletter **54**: p. 5-8.
2. Christianson, T.W., et al., *Multifunctional yeast high-copy-number shuttle vectors.* Gene, 1992. **110**(1): p. 119-22.
3. Klix, V., et al., *Functional characterization of MAT1-1-specific mating-type genes in the homothallic ascomycete Sordaria macrospora provides new insights into essential and nonessential sexual regulators.* Eukaryot Cell, 2010. **9**(6): p. 894-905.
4. Bloemendal, S., et al., *A homologue of the human STRIPAK complex controls sexual development in fungi.* Mol Microbiol, 2012. **84**(2): p. 310-23.
5. Pöggeler, S., et al., *Versatile EGFP reporter plasmids for cellular localization of recombinant gene products in filamentous fungi.* Curr Genet, 2003. **43**(1): p. 54-61.
6. Janus, D., et al., *An Efficient Fungal RNA-Silencing System Using the DsRed Reporter Gene.* Applied and Environmental Microbiology, 2007. **73**(3): p. 962-970.
7. Elleuche, S. and S. Pöggeler, *Visualization of peroxisomes via SKL-tagged DsRed protein in Sordaria macrospora.* Fungal Genetics Reports, 2008. **55**: p. 8-12.
8. Lichius, A. and N.D. Read, *A versatile set of Lifeact-RFP expression plasmids for live-cell imaging of F-actin in filamentous fungi* Fungal Genetics Reports 2010. **57**: p. 8-14.
9. Katoh, K., J. Rozewicki, and K.D. Yamada, *MAFFT online service: multiple sequence alignment, interactive sequence choice and visualization.* Briefings in Bioinformatics, 2019. **20**(4): p. 1160-1166.
